# Anoxic photochemical weathering of pyrite on Archean continents

**DOI:** 10.1126/sciadv.abn2226

**Published:** 2022-06-29

**Authors:** Jihua Hao, Winnie Liu, Jennifer L. Goff, Jeffrey A. Steadman, Ross R. Large, Paul G. Falkowski, Nathan Yee

**Affiliations:** 1 Department of Marine and Coastal Sciences, Rutgers University, New Brunswick, NJ 08901, USA.; 2 CAS Key Laboratory of Crust-Mantle Materials and Environments, School of Earth and Space Sciences, University of Science and Technology of China, Hefei 230026, China.; 3 Department of Earth and Planetary Sciences, Rutgers University, Piscataway, NJ 08854, USA.; 4 CODES, Centre for Ore Deposit and Earth Sciences, University of Tasmania, Hobart, TAS 7001, Australia.; 5 Department of Environmental Sciences, Rutgers University, New Brunswick, NJ 08901, USA.

## Abstract

Sulfur is an essential element of life that is assimilated by Earth’s biosphere through the chemical breakdown of pyrite. On the early Earth, pyrite weathering by atmospheric oxygen was severely limited, and low marine sulfate concentrations persisted for much of the Archean eon. Here, we show an anoxic photochemical mechanism of pyrite weathering that could have provided substantial amounts of sulfate to the oceans as continents formed in the late Archean. Pyrite grains suspended in anoxic ferrous iron solutions produced millimolar sulfate concentrations when irradiated with ultraviolet light. The Fe^2+^_(aq)_ was photooxidized, which, in turn, led to the chemical oxidation of pyritic sulfur. Additional experiments conducted with 2.68 Ga shale demonstrated that photochemically derived ferric iron oxidizes and dissolves sedimentary pyrite during chemical weathering. The results suggest that before the rise of atmospheric oxygen, oxidative pyrite weathering on Archean continents was controlled by the exposure of land to sunlight.

## Teaser

Experimental evidence shows that ultraviolet light can chemically break down pyrite to sulfate in ferruginous solutions.

## INTRODUCTION

Pyrite is the most abundant sulfide mineral and the primary source of sulfur in the geosphere. On the modern Earth, oxidative weathering of pyrite driven by atmospheric oxygen produces soluble sulfate ions that are assimilated by living matter. All three domains of life on Earth have evolved biological pathways to incorporate sulfate from the environment into their cells for the biosynthesis of the essential amino acids cysteine and methionine. Because photosynthetic cyanobacteria, algae, and higher plants are dependent on these assimilatory pathways (*1*–*3*), the sulfate released by pyrite weathering is critical for sustaining primary productivity and the production of oxygen on our planet (*1*, *2*).

During the Archean eon, Earth’s atmosphere contained little or no free oxygen (*3*, *4*), and pyrite weathering by O_2_ was severely limited (*5*, *6*). The planetary surface maintained anoxic conditions for nearly 2 billion years ago (Ga) until the Great Oxidation Event at about 2.4 Ga (*7*) as evidenced by mass-independent fractionation of sulfur isotopes (*8*). This anoxic environment resulted in the preservation of pyrite and other reduced minerals during the continental weathering and riverine transport (*9*, *10*). For much of the Archean eon, the formation of sulfate from the oxidative pyrite weathering by oxygen was negligible. Consequently, sulfate was a scarce nutrient in the Archean oceans, and the low concentrations of sulfate restricted biological productivity (*11*). Paradoxically, despite the anoxic conditions, an increase in the total sulfur weathering flux and the mobilization of transition metals from sulfide minerals have been observed in Neoarchean marine sedimentary records (*12*–*14*), suggesting that sulfide mineral oxidation on land occurred before the Great Oxidation Event. This apparent paradox questions the oxidative processes on Archean continents that could have mediated the pyrite weathering in the absence of atmospheric oxygen.

The ozone-free Archean atmosphere allowed the penetration of ultraviolet (UV) light that photooxidized Fe^2+^ (*15*–*21*), but very little is known about the impact of iron photogeochemistry on the early sulfur cycle. Cairns-Smith (*15*) suggested that irradiation of acidic waters by short UV light (200 to 300 nm) oxidized ferrous to ferric iron and formed hydrogen gas

$$2{\text{Fe}}^{2+}(\text{aq})+2H^{+}\to 2{\text{Fe}}^{3+}(\text{aq})+H_{2}$$ (1)

Braterman *et al.* (*16*) also noted that circumneutral photooxidation of the dissolved ferrous iron species Fe(OH)^+^_(aq)_ occurred with longer UV wavelengths between 300 and 450 nm (*16*). The high quantum yields of these photooxidation reactions indicate that sunlight played a fundamental role in shaping surficial environments in the Archean, including the production of Fe^3+^, the precipitation of iron oxides, and the reduction of H^+^ to H_2_.

Ferric iron is a strong oxidant that chemically oxidizes pyritic sulfur to sulfate (*22*)

$${\text{FeS}}_{2}(s)+14{\text{Fe}}^{3+}+8H_{2}O\to 15{\text{Fe}}^{2+}+2{{\text{SO}}_{4}}^{2‐}+16H^{+}$$ (2)

This reaction is kinetically favorable, with rates of pyrite oxidation by Fe^3+^ exceeding those of FeS_2(s)_ oxidation by O_2_ under acidic conditions (*23*). In addition to sulfate production, reaction 2 produces high levels of acidity. At low pH, the regeneration of Fe^3+^ is the rate-limiting step, and in modern acid mine drainage sites, Fe^2+^ oxidation is catalyzed by aerobic chemolithotrophic bacteria (*22*). Acid rock drainage also occurred on the early Earth (*24*), and aerobic bacteria have been implicated as the primary agents of pyrite weathering in the Neoarchean (*12*). Alternatively, Fe^3+^ might have been formed under anoxic conditions by photoferrotrophy (*25*) or abiotically by Fe^2+^ photooxidation (*15*, *16*). To date, the role of photochemically derived Fe^3+^ in pyrite weathering remains poorly understood.

In this study, we conducted photochemistry experiments to investigate the oxidative dissolution of pyrite in anoxic ferruginous waters. The objective was to determine whether UV light oxidizes pyritic sulfur to sulfate through Fe^3+^ derived from the photooxidation of dissolved ferrous iron. The geologic relevance of this photochemical process was further investigated by conducting photooxidation experiments with 2.68 Ga pyritic shale to simulate chemical weathering of pyrite-bearing Archean rocks. A photogeochemical model was developed to estimate the global amounts of sulfate that could have been produced by photochemical weathering. The results demonstrate that pyrite weathering can be driven by UV light under strict anoxic conditions, thus revealing a previously unrecognized mechanism of sulfate formation on the early Earth.

## RESULTS

**Fig. 1.**
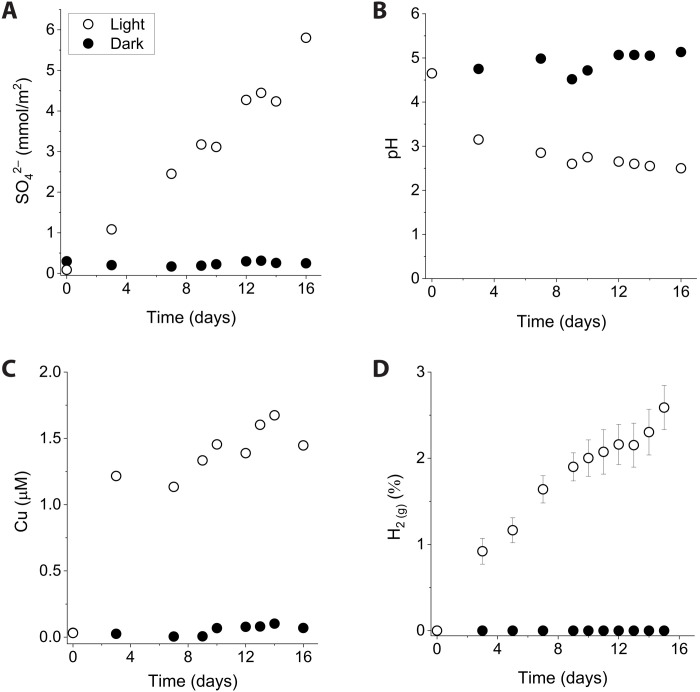
Oxidative dissolution of pyrite during Fe^2+^_(aq)_ photooxidation. Pyrite grains (0.32 m^2^/liter) were suspended in an anoxic solution containing 1.5 mM Fe^2+^ and irradiated with UV light (open circles). Dark control experiments were conducted in reaction vessels wrapped in aluminum foil (closed circles). (**A**) Production of sulfate. (**B**) Acidification of the water. (**C**) Release of copper. (**D**) Formation of H_2_.

Pyrite grains suspended in anoxic ferrous iron solutions (1.5 mM) produced sulfate when irradiated with UV light (Fig. 1A). Normalized to surface area, the rate of sulfate formation was 3.8 × 10^−9^ ± 0.2 × 10^−9^ mol/m^2^ per second at a UV photon flux of 1.8 × 10^21^ photons/s per square meter. Approximately 1.8 mM sulfate was produced after 16 days. Sulfate production was also observed with a long-pass filter that blocks wavelengths that can lead to water photolysis (<190 nm), thus ruling out the involvement of photochemically induced reactive oxygen species in pyrite oxidation (fig. S1). Acidification of the water was concurrent to the production of sulfate (Fig. 1B). The pH of the pyrite suspension decreased from 4.7 to 2.5. After 1 week of irradiation, dissolved Fe^3+^_(aq)_ was detectable in the aqueous phase with concentrations ranging between 0.2 and 0.3 mM, which closely matched the solubility of ferrihydrite (*TOT*[Fe^3+^_(aq)_]_ferrihydrite_ = 0.28 mM at pH 2.5). X-ray diffraction (XRD) analysis of the irradiated solids did not show any crystalline iron oxide phases other than the residual unreacted pyrite (fig. S2). In addition to the formation of sulfate, pyrite dissolution resulted in the release of soluble copper (Cu) (Fig. 1C). Up to 1.7 μM dissolved Cu was measured in filtered samples. Cu concentrations increased rapidly early in the irradiation and then slowed due to possible readsorption onto poorly crystalline secondary precipitates. During the irradiation, substantial amounts of H_2_ was also continually produced from Fe^2+^ photooxidation (Fig. 1D). By the end of the experiment, the headspace gas composition reached 2.3% H_2_. The formation of sulfate, decrease in pH, release of copper, and production of hydrogen gas were not observed in dark controls.

**Fig. 2.**
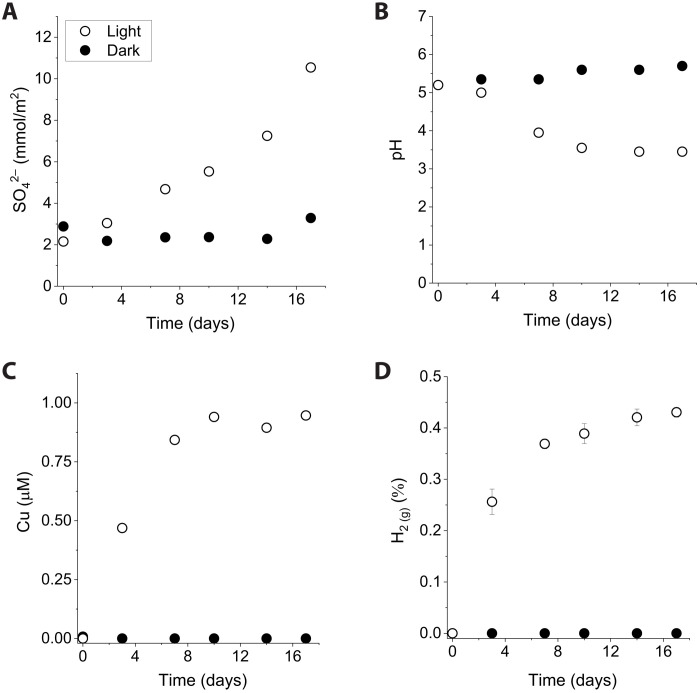
Oxidative weathering of 2.65 Ga Oroya shale during Fe^2+^_(aq)_ photooxidation. UV irradiation experiments (open circles) and dark controls (closed circles) were conducted with crushed rock (pyrite surface area of 0.03 m^2^/liter) suspended in anoxic Fe^2+^_(aq)_ solutions (0.2 mM). (**A**) Production of sulfate. (**B**) Acidification of the water. (**C**) Release of copper. (**D**) Formation of H_2_.

**Fig. 3.**
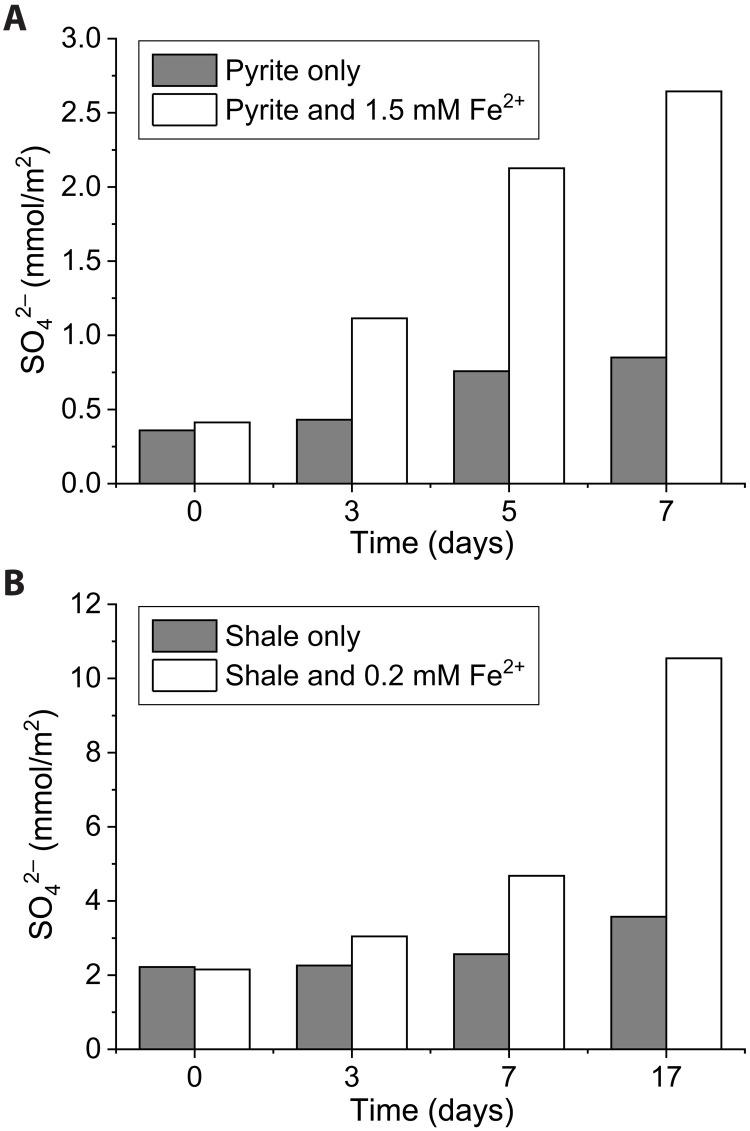
The effect of Fe^2+^_(aq)_ on photochemical pyrite oxidation. Sulfate production in UV irradiation experiments with Fe^2+^ (white bars) and without Fe^2+^ (gray bars) in experiments with (**A**) pyrite and (**B**) Oroya shale.

To test whether this photochemical reaction could have been a geologically relevant process on the early Earth, we repeated our experiments with 2.68 Ga pyritic shale from the Kalgoorlie sequence, Western Australia (fig. S3) at Fe^2+^ concentrations that mimicked Archean river water conditions. Irradiation of the pyritic shale in ferrous iron solutions (0.2 mM) produced sulfate at a rate of 4.2 × 10^−9^ ± 0.2 × 10^−9^ mol/m^2^ per second (Fig. 2A) concurrent to a decrease in solution pH from 5.2 to 3.5 (Fig. 2B). In addition to pyrite, the mineral components of the shale determined by powder XRD included quartz, ankerite, siderite, and muscovite, which buffered the solution pH. After 17 days, Cu was released and accumulated to a dissolved concentration of 0.9 μM (Fig. 2C), and the headspace gas composition reached 0.4% H_2_. Parallel pyrite irradiation experiments conducted without adding Fe^2+^ to the solutions resulted in limited sulfate production (Fig. 3).

**Fig. 4.**
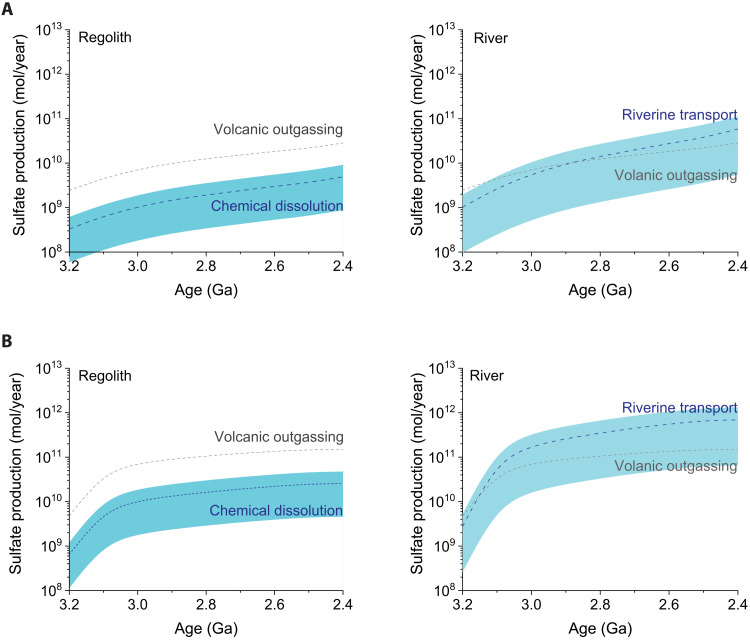
Sulfate production on Archean weathering environments due to the oxidative dissolution of pyrite by photooxidized Fe^2+^. Blue dashed lines represent the average estimates, and the blue envelope represents the range of sulfate production values based on the upper and lower bounds of possible Archean environmental parameters. Gray dashed lines represent the estimated flux from volcanic outgassing. (**A**) Calculations based on land exposure reported by Flament *et al.* (*27*). (**B**) Calculations based on the rapid continental growth scenario of Korenaga *et al.* (*28*). Shown on the left is sulfate production in Archean regolith. Shown on the right is sulfate production in Archean rivers (*28*).

An Archean photogeochemical model was developed to constrain the rates of sulfate production from pyrite oxidation by photochemically derived Fe^3+^. On the basis of the solar flux at the Earth’s surface (*26*) and recent measurements of Fe^2+^ photooxidation quantum yields (*20*), we found that anoxic photochemical weathering of pyrite was kinetically favorable in both Archean regolith and rivers (Fig. 4). Using the continental growth models of either Flament *et al.* (*27*) or Korenaga *et al.* (*28*), our calculations indicated that sulfate production increased over time until the end of Archean eon when stratospheric ozone attenuated the atmospheric transmission of UV photons (*8*). The extent of sulfate production was dependent on pH, Fe^2+^ concentration, pyrite surface area, and light penetration depth. Assuming that weathering fluids buffered at pH 6 and [Fe^2+^] = 100 μM (*29*–*31*), we conservatively estimate 0.5 × 10^10^ to 2.6 × 10^10^ mol/year of sulfate production in regolith by the end of Archean eon. The range of sulfate production values in the regolith was mainly dependent on the penetration depth of UV light, which was set to be 0.1 to 1 mm (*32*). In the Archean rivers, pyrite dissolution was much more extensive than in regolith and contributed 0.6 × 10^11^ to 6.9 × 10^11^ mol/year of sulfate production by 2.4 Ga. Continental erosion rates and pyrite transport times were the main parameters affecting riverine sulfate fluxes. Assuming a pyrite transport time of 1 ka, approximately 0.36% of the total suspended pyrite in the riverine system was dissolved. Our estimates of global sulfate production from photochemical pyrite weathering were strongly dependent on land emergence patterns in the Archean eon (tables S1 and S2). The slow continental growth model proposed by Flament *et al.* (*27*) (Fig. 4A) yielded lower sulfate fluxes compared to the fast continental growth model of Korenaga *et al.* (*28*) (Fig. 4B)*.*

## DISCUSSION

The results of this study offer an alternative explanation that reconciles the evidence of oxidative pyrite weathering and atmospheric anoxia on the Archean Earth. Oxidative weathering records using transition metals and their isotopes suggest that crustal sulfide weathering occurred in the Archean before the Great Oxidation Event (*13*, *33*). These observations have been difficult to interpret because there was little or no free oxygen in the Archean atmosphere (*34*), and oxygen is thought to have been required to chemically weather sulfide minerals (*3*, *4*, *35*, *36*). Previous studies have suggested local production of O_2_ by terrestrial cyanobacteria (*37*) and subaerial microbial mats (*38*), which may have facilitated either aerobic microbial pyrite oxidation or abiotic oxidation under low O_2_ conditions. Stüeken *et al.* (*12*) proposed that the pyrite oxidation on land in the Neoarchean was mediated by microbes using oxygen. Recently, Johnson *et al.* (*39*) suggested that the dissolution of sulfide minerals and mobilization of chalcophile elements in the Archean could proceed at partial pressure of oxygen (*p*O_2_) > 10^–6.9^ present atmospheric level. Using Mo concentration data in the Archean sedimentary rocks, Johnson *et al.* (*39*) estimated a flux of ≥6 × 10^10^ mol of O_2_ production per year during the late Archean. If we assume that oxidative weathering of pyrite was mediated by oxygen (FeS_2_ + 7/2 O_2_ + H_2_O → Fe^2+^ + 2SO_4_^2−^ + 2H^+^), then this O_2_ flux would correspond to a sulfate production rate of 3.4 × 10^10^ mol/year, which is significantly less than the global sulfate flux of 5.5 × 10^11^ mol/year reconstructed from marine sedimentary archives (*12*). This suggests that additional oxidants were involved in Archean pyrite weathering.

At low *p*O_2_, the rates of pyrite oxidation by Fe^3+^ are expected to greatly exceed that of FeS_2(s)_ oxidation by oxygen-dependent mechanisms. Our experimental results demonstrated that UV light can sustain the production of Fe^3+^ and drive the oxidative dissolution of pyrite without oxygen. Therefore, oxidative weathering and the release of chalcophile elements from sulfides could have occurred in an anoxic Archean atmosphere via mineral oxidation by photochemically derived Fe^3+^. We suggest that the pyrite dissolution observed in our experiment was mediated by the photooxidation of both dissolved and adsorbed Fe^2+^. The aqueous speciation of ferrous iron was dominated by free Fe^2+^, which was photooxidized to ferric iron by UV light. Once formed, the aqueous ferric ions attacked the mineral surface and chemically oxidized the pyritic sulfur. The oxidized sulfur then reacted with the oxygen atoms in water molecules to form sulfate (*40*, *41*). In addition, Fe^2+^ adsorbed at the pyrite mineral-water interface may have also been photooxidized. Because the bandgap of pyrite is only 0.95 eV (*42*), UV and visible light could have excited an electron within the mineral, causing the oxidation of adsorbed Fe^2+^. In this scenario, the surface-bound Fe^3+^ accepts electrons from sulfide bonds and was cycled between oxidized and reduced states, acting as a conduit for electron transfer to protons producing H_2_.

We note that H_2_ production from both dissolved and adsorbed Fe^2+^ would have contributed to the global hydrogen cycle on the Archean Earth. In pyritic photogeochemical systems, the dissolution of pyrite could have continuously released Fe^2+^ into weathering fluids, allowing for sustained iron photooxidation. Kim *et al.* (*21*) previously estimated the hydrogen flux in ferruginous Archean oceans; however, H_2_ production from iron photooxidation on Archean continents remains poorly constrained. Further investigation is merited as terrestrial iron photooxidation and atmospheric escape of H_2_ would have influenced the redox state of the planetary surface over Archean time.

The photogeochemical modeling results indicated that the exposure of land to sunlight was an important factor that controlled Archean pyrite weathering. Our estimates of light-driven pyrite weathering (Fig. 4) are consistent with the reconstructed flux of continentally delivered sulfate using marine sedimentary records, which shows an increase in sedimentary sulfur from the middle to late Archean (*12*). Similarly, our model shows that continent formation and the growth of sun-exposed land increased sulfate delivery to the oceans from 3.2 to 2.4 Ga. The estimated sulfate production in rivers is significantly higher than in the regolith, with the extent of riverine pyrite weathering strongly dependent on river water chemistry and the duration of pyrite transport. Archean river water is estimated to accumulate up to tenths of millimolar of dissolved iron (*29*), which is similar to the concentration of FeCl_2_ used in the Archean shale irradiation experiment that produced up to 0.4 mM sulfate in our experiments. The rates of pyrite oxidation by Fe^3+^ in circumneutral waters from pH 5 to 8 are very similar (*23*) but accelerate markedly under acidic conditions. In our model, we assumed that the rivers were in equilibrium with Archean atmospheric gases (*29*, *31*) and buffered at pH 6. Lower riverine pH conditions may have existed if extensive pyrite oxidation occurred regionally, as pyrite weathering would have generated substantial amounts of acid. Furthermore, we found that the duration of pyrite transport in riverine systems would have affected sulfate production and the breakdown of pyrite grains. The transport time of suspended pyrite in rivers has been reported to vary from thousands to ten thousands of years depending on the length of rivers as well as relief and climate (*5*). Our calculations indicated that longer transport times would have promoted near-quantitative pyrite dissolution, while shorter transport times favored the preservation of detrital pyrite grains in the millimeter size range, which have been found in Archean sediments (*5*, *43*).

In local environments, anoxic photochemical pyrite weathering could have produced high sulfate concentrations, leading to the large S mass-dependent isotope fractionations observed in Archean samples. Under higher sulfate conditions, microbial sulfate reduction results in S isotope fractionations up to 45 per mil (‰) (*44*), and several Archean deposits exhibit large S isotope fractionations, suggesting locally high sulfate concentrations (*45*, *46*). For example, the pyrite in the Hemlo gold deposit in Wawa, Ontario, shows δ^34^S values ranging from −17.5 to +12.6‰ (*45*), and the Dresser Formation in North Pole (*46*) and the Moodies Group in Barberton Greenstone Belt, Africa, show fractionations up to 21.1 and 34‰, respectively (*47*). All three of these locations also contain sulfate minerals such as barite and anhydrite. The S isotope fractionations and the occurrence of sulfate minerals suggest that geologic settings such as restricted basins and shallow water environments accumulated high sulfate concentrations even under the low *p*O_2_ conditions of the Archean atmosphere (*45*, *46*). These types of environments could have had extensive Fe^3+^-mediated pyrite dissolution due to the shallow water depth that allowed light penetration and Fe^2+^ photooxidation. Moreover, Archean pyrites are known to contain mass-independent fractionation S isotope signals (*7*, *8*, *14*, *47*, *48*), and the dissolution of these pyrites should release sulfate with Δ^33^S anomalies inherited from the pyritic sulfur. Currently, it is unknown whether UV-driven sulfate production from pyrite oxidation causes additional isotope fractionations that might be discernable in the Archean geologic record.

On a global scale, our calculations indicated that the production of sulfate by photochemical pyrite weathering was comparable to the sulfate flux by volcanic outgassing (Fig. 4). The continental growth models of Flament *et al.* and Korenaga *et al.* (*27*, *28*) yielded total pyrite weathering estimates of 6.4 × 10^10^ and 7.2 × 10^11^ mol/year, respectively, and volcanic outgassing fluxes of 2.8 × 10^10^ and 1.5 × 10^11^, respectively (tables S1 and S2). Mantle cooling tempered the extent of volcanic activity in the late Archean (*6*, *49*, *50*), while the growth of continental land masses increased the production of sulfate from pyrite weathering. By 2.4 Ga, we estimate that sulfate production from photochemical pyrite weathering could have been equal to or greater than the sulfate flux from volcanic outgassing. Our results call for the revision of sulfur mass balance models to account for photochemical pyrite weathering as a major source of sulfate to the Archean biosphere.

## MATERIALS AND METHODS

### Pyrite irradiation

Pyrite was purchased from Sigma-Aldrich (CAS#778117) and washed with 1 M HCl, deoxygenated Milli-Q water (four times) and 95% ethanol. Washed pyrite grains were then dried under anoxic conditions at ambient temperature and resuspended in deoxygenated Milli-Q water. The Brunauer-Emmett-Teller (BET) surface area of the pyrite grains was 0.4656 m^2^/g. The Cu content of the pyrite was 314 parts per million (ppm). The pyrite suspension, Milli-Q water, chemical reagents, glassware, and plastic magnetic stirrers were kept in an anaerobic chamber before experimentation to minimize oxygen contamination. UV irradiation experiments were conducted by mixing the pyrite suspension with a ferrous chloride solution in N_2_-purged quartz reaction cells at concentrations of 0.68 g/liter of pyrite and 1.5 mM FeCl_2_. We set the initial concentration of Fe^2+^_(aq)_ in these experiments to solubility of siderite (FeCO_3_) under mildly acidic conditions ([Fe^2+^] = 10^–2.8^ M at pH 6) because the maximum concentration of Fe^2+^_(aq)_ in Archean weathering environments was likely controlled by the siderite solubility (*5*, *10*, *29*, *51*, *52*). A 450-W Hg vapor lamp (Hanovia PC451.050) in a photochemical quartz immersion well was used to irradiate the samples. The reaction cells were sealed with gas-impermeable butyl rubber stoppers and aluminum crimp seals to maintain strict anoxic conditions. The pyrite grains were continuously suspended with magnetic stirrers and irradiated for 16 days. Dark controls were wrapped in aluminum foil and stirred for the same duration of time.

At periodic intervals, the headspace gas and mineral suspension were sampled with a needle and a syringe. To remove O_2_, the needle and the syringe were purged with N_2_ gas multiple times before sampling. The concentration of H_2_ in headspace samples was measured by gas chromatography (GC) with a thermal conductivity detector (model 310, SRI Instruments). GC was also used to analyze O_2_ to ensure that there was no atmospheric contamination. No oxygen contamination was detected in any of the samples, indicating that strict anoxic conditions were maintained throughout the experiment. Aliquots of the mineral suspension were removed and filtered (0.2 μm) for chemical analysis. The reaction cell was agitated to homogenize the suspension when sampling to maintain a constant solution:solid ratio. The pH was measured using a handheld HANNA pH probe in an anaerobic glove box. Sulfate concentrations of filtered aliquots were determined using a Dionex Aquion Ion Chromatography System (Thermo Fisher Scientific) equipped with a Dionex IonPac AS-9 HC column. Acidified filtrate samples (2% HNO_3_) were analyzed using an iCAP 7400 Inductively Coupled Plasma Optical Emission Spectrometer (Thermo Fisher Scientific) to determine the concentration of Cu at 324.754 nm. Aqueous iron concentrations were measured at each time point using the ferrozine assay, and [Fe^3+^_(aq)_] was calculated as the difference between the *TOT*[Fe]_Dissolved_ and [Fe^2+^_(aq)_]. Powder XRD analysis was conducted on the solids using a Rigaku MiniFlex 6G equipped with a Co anode (λ = 1.790 Å).

### Irradiation of pyritic shale

To further investigate pyrite weathering in the late Archean, UV irradiation experiments were conducted with 2.68 Ga pyritic shale. We selected pyrite-bearing shale for this experiment because the emergence of Neoarchean crust above sea level (*27*, *28*, *53*–*55*) would have exposed large areas of marine sedimentary rocks for chemical weathering. The Oroya shale sample used in this study (GMSP-009) was collected at the Golden Mile Super Pit, Kalgoorlie, Western Australia (fig. S3) (*48*). It contained thin beds of fine-grained pyritic shale, except for a ~25-mm-thick layer of colloform pyrite intergrown with carbonate. The portion of GMSP-009 analyzed in this study contained 12.2% sulfur and 348 ppm of Cu. GMSP-009 also contained Co, Ni, As, Se, Mo, Ag, Sn, Sb, Te, Au, Hg, Tl, Pb, and Bi. Before experimentation, the rock was crushed, sieved, and washed three times with deoxygenated Milli-Q water. Mineral components of the rock were analyzed using powder XRD. The BET surface area was 3.0172 m^2^/g. UV irradiation of the crushed rock was performed using the same experimental setup as pyrite experiments. The concentrations of shale and FeCl_2_ were 0.07 g/liter and 0.2 mM, respectively. Lower Fe^2+^ concentrations were used in these experiments to better mimic Archean river water (*29*). At periodic intervals, the headspace gas was sampled for H_2_ analysis, and aliquots of the solid suspension were removed and filtered for pH, sulfate, and dissolved metal analysis using the analytical methods described above.

### Archean photogeochemical model

We estimated the sulfate production from Fe^3+^-oxidative pyrite dissolution by considering two Archean continental weathering environments: (i) Fe^3+^ limited in regolith and (ii) pyrite limited in rivers.

For the regolith calculation, equations from Anbar and Holland (*17*) were adopted to estimate the photooxidation flux of Fe^2+^ in the surface layers of regolith

$$\begin{array}{c} \theta_{\text{Fe}}=(3.15\times 10^{7}s\;{\text{year}}^{-1})(55.8\times 10^{3}\text{mg}\;{\text{mol}}^{-1})\times \frac{0.5[{\text{Fe}}^{2+}]}{6.02\times 10^{23}}\\ \int_{0}^{z}\int_{200\text{nm}}^{400\text{nm}}\phi^{\text{Fe}}\varepsilon^{\mathrm{Fe}}F(\lambda ,z)d\lambda \mathrm{dz} \end{array}$$ (3)

where

$$F(\lambda ,z)=F_{0}10^{-\mathrm{kz}/\text{cosω}}$$ (4)

Details of model parameters are explained by Anbar and Holland (*17*). Briefly, [Fe^2+^] is the steady-state concentration of dissolved ferrous iron in the water; ϕ^Fe^ is the quantum yield of Fe^2+^ photooxidation; *F*_0_ is the photon flux at the Earth’s surface; *z* is the penetration depth; ω is the incident angle of the radiation set at ω = 35° (*56*). The wavelength of UV light (λ) was between 200 and 400 nm, representing the range compensating the shielding effect for <200 nm by the CO_2_-rich atmosphere. We assumed that the regolith was wet, and a dissolved ferrous iron concentration [Fe^2+^] = 0.1 mM was used in accordance with previous Archean weathering studies (*29*–*31*). This Fe^2+^ concentration is comparable to dissolved ferrous iron values measured in modern anoxic groundwater (*57*, *58*). The vadose zone in Archean regolith likely experienced frequent wet-dry cycles (*59*), which may have dissolved Fe^2+^ from bedrock or delivered ferrous iron from the groundwater. The penetration depth of UV light (*z*) in regolith was set to be 0.1 to 1 mm (*32*). The quantum yield of the Fe^2+^ photooxidation (ϕ^Fe^) was estimated using the equation reported by Tabata *et al.* (*20*)

$$\phi =0.103+2.17\times {\left[H^{+}\right]}^{0.5}$$ (5)

A pH value of 6 was used for the weathering fluid (*29*). We used the molar absorptivity (ϵ) of Fe^2+^ from Anbar and Holland (*17*) and the photon flux at the Earth’s surface (*F*_0_) from Claire *et al.* (*26*) (http://depts.washington.edu/naivpl/content/models/solarflux/).

We assumed that continental weathering eroded the land at a rate of approximately 0.1 to 2 km/Ma (corresponding to 0.1 to 2 mm/year) (*60*) and that the pyrite content was 621 μg/g in the upper continental crust (*61*). The steady-state amount of pyrite in riverine systems was estimated on the basis of the area of exposed land during the Archean eon. One calculation was performed using the continental growth model of Flament *et al.* (*27*), and the second calculation used the land area reported by Korenaga *et al.* (*28*). A pyrite grain radius of 5 mm was selected, consistent with the observations that the Archean detrital pyrites are commonly in the millimeter size range (*43*). In modern riverine systems, the transport time of suspended pyrite has been reported to vary from thousands to ten thousands of years depending on the length of rivers as well as relief and climate (*5*). Here, we chose to use a modern transport time (*t*_Modern_) of 1000 years, as a conservative estimate for pyrite weathering. Because river length is proportional to land width, we used the following relationship to estimate the riverine transport time on Archean continents (*t*_Archean_)

$$t_{\text{Archean}}={t_{M\text{odern}}}^{*}\;\sqrt{f}$$ (6)

where *f* is the fraction (*S*_Archean_/*S*_Modern_) of Archean land area (*S*_Arcehan_) relative to the modern (*S*_Modern_) based on the work of Flament *et al.* (*27*) and Korenaga *et al.* (*28*). To account for the riverine transport time of eroded pyrite grains from different parts of river systems (e.g., headwaters or middle or near coastal areas), we averaged the transport time by dividing *t*_Archean_ by 2.

To calculate the rates of pyrite oxidation to sulfate, we adopted the kinetic law from Williamson and Rimstidt (*62*) and extrapolated the equation to pH 6

$$r_{{\text{FeS}}_{2}}=\frac{k{\left[\text{Fe}(\text{III})\right]}^{0.3}}{{\left[H^{+}\right]}^{0.32}{\left[{\text{Fe}}^{2+}\right]}^{0.47}}$$ (7)

where *r*_FeS2_ is the rate of pyrite destruction in moles per square meter per second, and *k* = 10^–8.58^ under ambient conditions. Sulfate production rates were determined by assuming quantitative conversion of pyritic sulfur to sulfate. The concentration of Fe^3+^ in the river water was constrained by the solubility of ferrihydrite at pH 6, which was calculated by SUPCRT92b. To test whether the rate law is applicable to our systems, we calculated theoretical sulfate production rates using Eq. 7 for the experimental conditions in the pyrite and shale irradiation experiments. The predicted sulfate production rates based on the Williamson and Rimstidt model for the pyrite and shale experiments are 0.15 and 0.016 mM per day, respectively, which closely match our experimentally determined rates of 0.12 and 0.018 mM per day, respectively.

### Sulfate production from volcanic outgassing

We chose to adopt the same method by Canfield (*6*) and later by Stüeken *et al.* (*12*) to estimate the flux of sulfate by volcanic outgassing. A dominant sulfur species from volcanic gas is SO_2_, which could disproportionate into SO_4_^2^ in surface water

$$3{\text{SO}}_{2}+3H_{2}O\to 2{\text{HSO}}_{4^{-}}+S+2H^{+}$$ (8)

The annual flux of SO_2_ from modern volcanoes (*F*_mod_) is estimated to be 4 × 10^12^ g/year (*63*), corresponding to 1.25 × 10^11^ mol/year. Following previous studies, we assume that the volcanic outgassing flux (*F_t_*) at time *t* is proportional to volcanic activity coefficient (*Q_t_*) and land area (*S_t_*) (*31*)

$$F_{t}={F_{\text{mod}}}^{*}{Q_{t}}^{*}S_{t}$$ (9)

According to Canfield (*6*), *Q_t_* = 1.00 + 0.1217*t + 0.0924*t^2^ (*t* in billion years).
