# Supplementary material for: Anoxic photochemical weathering of pyrite on Archean continents
Source: Sci Adv. 2022 Jun 29;8(26):eabn2226. doi: 10.1126/sciadv.abn2226 (PMC9242442; doi:10.1126/sciadv.abn2226)
Supplement: Supplementary file 1 — Figs. S1 to S3 Tables S1 and S2 [file sciadv.abn2226_sm.pdf]

Science Advances
AAAS

Supplementary Materials for

Anoxic photochemical weathering of pyrite on Archean continents

Jihua Hao *et al.*

Corresponding author: Nathan Yee, nyee@envsci.rutgers.edu

Sci. Adv. **8**, eabn2226 (2022)
DOI: 10.1126/sciadv.abn2226

This PDF file includes:

Figs. S1 to S3
Tables S1 and S2

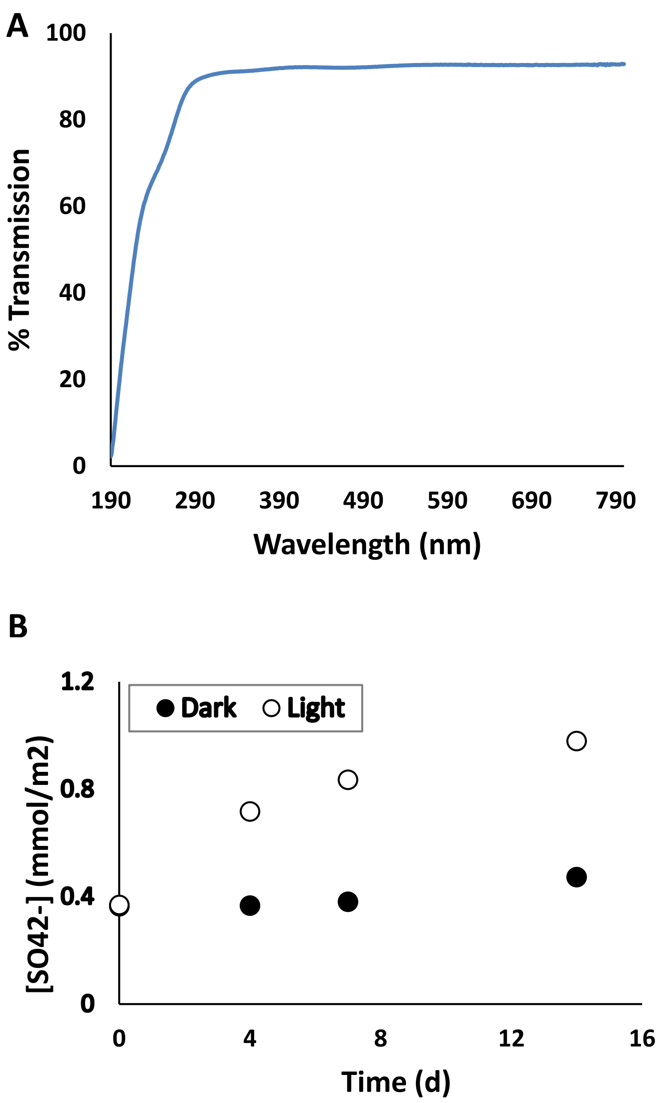

Fig. S1. Sulfate production in the longpass filter experiment. Pyrite grains suspended in solution were irradiated with light that was filtered by a WG 225 longpass filter. (**A**) Transmission spectrum of the WG 225 longpass filter. The wavelengths involved in water photolysis 140-190 nm are blocked by the longpass filter. (**B**) The production of sulfate during pyrite irradiation (open circles). The experimental setup with the longpass filter diminished the overall intensity of transmitted light by approximately 4-5 folds resulting in lower rates of sulfate formation. No sulfate production was observed in dark controls (closed circles).

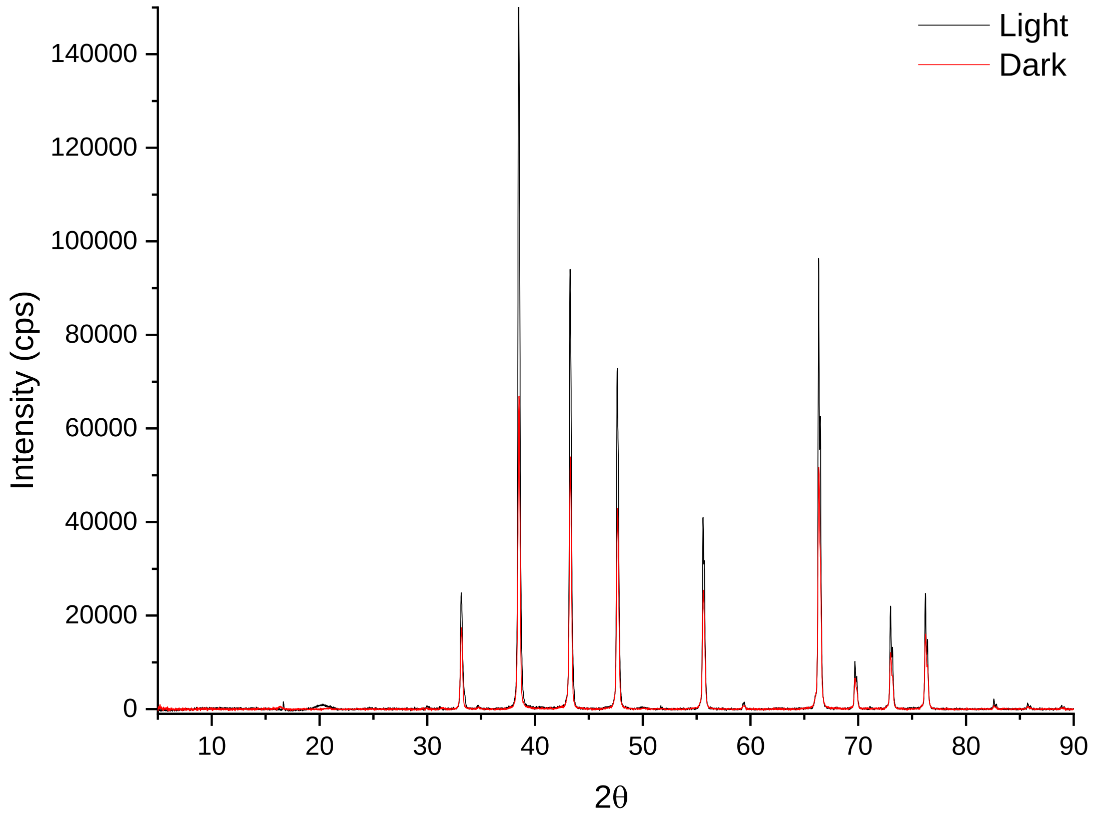

Fig S2. X-ray diffractograms of pyrite grains after UV irradiation experiment (black) and dark controls (red). Samples were analyzed using a Rigaku MiniFlex 6G equipped with a Co anode (λ = 1.790 Å). The diffraction peaks correspond to the pyrite d-spacings 3.13, 2.71, 2.42, 2.21, 1.92, 1.63, 1.56, 1.50, and 1.45 Å.

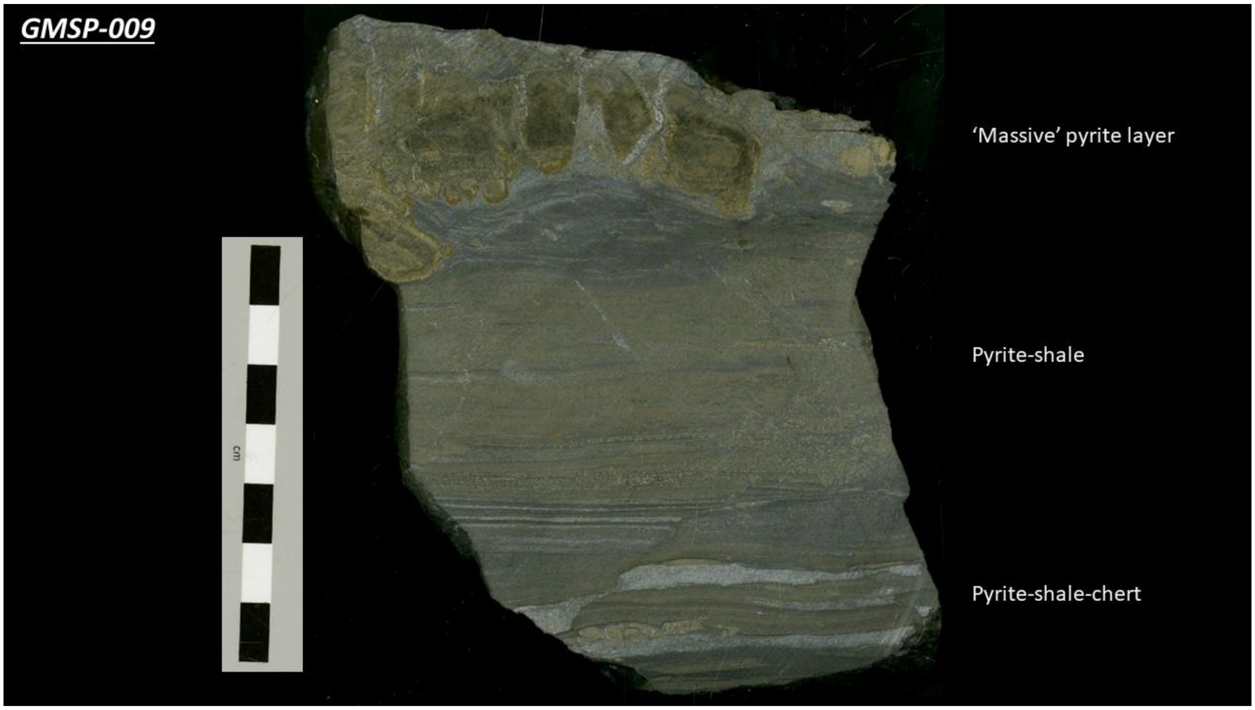

Fig. S3. The Oroya Shale sample (GMSP-009) used the irradiation experiment. The sample is characterized by thin beds of chert alternating with mm-scale pyrite-rich laminae. Pyrite in these layers is generally fine- to medium-grained, but rare cm-scale nodules or lenses are also present. Toward the top of the sample, the pyrite morphology changes from fine-grained and disseminated to colloform and brecciated. Pyrite-poor laminae are present immediately below this colloform pyrite zone. Small micro-faults are also visible throughout the sample; these manifest as bedding offsets. The trace element and S isotope chemistry of pyrite in GMSP-009 are reported in Steadman *et al*. (*39*). The sample was crushed, sieved, and washed before the irradiation experiment.

Age (Ga)	Regolith Lower (moles/yr)	Regolith Upper (moles/yr)	Regolith Average (moles/yr)	River Lower (moles/yr)	River Upper (moles/yr)	River Average (moles/yr)	Total Weathering (moles/yr)	Volcanic outgassing (moles/yr)
3.2	6.04E+07	6.04E+08	3.32E+08	9.73E+07	1.95E+09	1.02E+09	1.35E+09	2.45E+09
3.1	1.16E+08	1.16E+09	6.40E+08	2.60E+08	5.21E+09	2.73E+09	3.37E+09	4.58E+09
3	1.88E+08	1.88E+09	1.03E+09	5.34E+08	1.07E+10	5.61E+09	6.64E+09	7.17E+09
2.9	2.69E+08	2.69E+09	1.48E+09	9.18E+08	1.84E+10	9.63E+09	1.11E+10	9.97E+09
2.8	3.50E+08	3.50E+09	1.92E+09	1.36E+09	2.71E+10	1.42E+10	1.62E+10	1.25E+10
2.7	4.35E+08	4.35E+09	2.39E+09	1.88E+09	3.77E+10	1.98E+10	2.22E+10	1.51E+10
2.6	5.46E+08	5.46E+09	3.00E+09	2.64E+09	5.29E+10	2.78E+10	3.08E+10	1.84E+10
2.5	6.62E+08	6.62E+09	3.64E+09	3.53E+09	7.06E+10	3.71E+10	4.07E+10	2.16E+10
2.4	8.98E+08	8.98E+09	4.94E+09	5.58E+09	1.12E+11	5.86E+10	6.36E+10	2.84E+10

Table S1. Sulfur flux estimates from the photogeochemical model. The land area used in this calculation was based on the continental growth model of Flament *et al.* (*27*).

Age (Ga)	Regolith Lower (moles/yr)	Regolith Upper (moles/yr)	Regolith Average (moles/yr)	River Lower (moles/yr)	River Upper (moles/yr)	River Average (moles/yr)	Total Weathering (moles/yr)	Volcanic outgassing (moles/yr)
3.2	1.17E+08	1.17E+09	6.45E+08	2.63E+08	5.27E+09	2.77E+09	3.41E+09	4.76E+09
3.1	1.11E+09	1.11E+10	6.12E+09	7.71E+09	1.54E+11	8.09E+10	8.70E+10	4.39E+10
3	1.89E+09	1.89E+10	1.04E+10	1.71E+10	3.42E+11	1.79E+11	1.90E+11	7.23E+10
2.9	2.43E+09	2.43E+10	1.34E+10	2.49E+10	4.98E+11	2.61E+11	2.75E+11	9.01E+10
2.8	2.96E+09	2.96E+10	1.63E+10	3.33E+10	6.67E+11	3.50E+11	3.66E+11	1.06E+11
2.7	3.50E+09	3.50E+10	1.92E+10	4.29E+10	8.58E+11	4.51E+11	4.70E+11	1.22E+11
2.6	4.02E+09	4.02E+10	2.21E+10	5.28E+10	1.06E+12	5.54E+11	5.76E+11	1.35E+11
2.5	4.52E+09	4.52E+10	2.49E+10	6.31E+10	1.26E+12	6.63E+11	6.87E+11	1.48E+11
2.4	4.65E+09	4.65E+10	2.56E+10	6.58E+10	1.32E+12	6.91E+11	7.17E+11	1.47E+11

Table S2. Sulfur flux estimates from the photogeochemical model. The land area used in this calculation was based on the continental growth model of Korenaga *et al.* (*28*)
